# Cartilage regeneration using improved surface electrospun bilayer polycaprolactone scaffolds loaded with transforming growth factor-beta 3 and rabbit muscle-derived stem cells

**DOI:** 10.3389/fbioe.2022.971294

**Published:** 2022-08-23

**Authors:** Mantas Malinauskas, Lina Jankauskaite, Lauryna Aukstikalne, Lauryna Dabasinskaite, Augustinas Rimkunas, Tomas Mickevicius, Alius Pockevicius, Edvinas Krugly, Dainius Martuzevicius, Darius Ciuzas, Odeta Baniukaitiene, Arvydas Usas

**Affiliations:** ^1^ Institute of Physiology and Pharmacology, Lithuanian University of Health Sciences, Kaunas, Lithuania; ^2^ Faculty of Chemical Technology, Kaunas University of Technology, Kaunas, Lithuania; ^3^ Department of Veterinary Pathobiology, Veterinary Academy, Lithuanian University of Health Sciences, Kaunas, Lithuania

**Keywords:** cartilage regeneration, PCL scaffolds, ozone treatment, transforming growth factor-beta 3, rabbit MDSCs, cell-scaffold construct

## Abstract

Polycaprolactone (PCL) has recently received significant attention due to its mechanical strength, low immunogenicity, elasticity, and biodegradability. Therefore, it is perfectly suitable for cartilage tissue engineering. PCL is relatively hydrophobic in nature, so its hydrophilicity needs to be enhanced before its use in scaffolding. In our study, first, we aimed to improve the hydrophilicity properties after the network of the bilayer scaffold was formed by electrospinning. Electrospun bilayer PCL scaffolds were treated with ozone and further loaded with transforming growth factor-beta 3 (TGFβ3). *In vitro* studies were performed to determine the rabbit muscle-derived stem cells’ (rMDSCs) potential to differentiate into chondrocytes after the cells were seeded onto the scaffolds. Statistically significant results indicated that ozonated (O) scaffolds create a better environment for rMDSCs because collagen-II (Coll2) concentrations at day 21 were higher than non-ozonated (NO) scaffolds. In *in vivo* studies, we aimed to determine the cartilage regeneration outcomes by macroscopical and microscopical/histological evaluations at 3- and 6-month time-points. The Oswestry Arthroscopy Score (OAS) was the highest at both mentioned time-points using the scaffold loaded with TGFβ3 and rMDSCs. Evaluation of cartilage electromechanical quantitative parameters (QPs) showed significantly better results in cell-treated scaffolds at both 3 and 6 months. Safranin O staining indicated similar results as in macroscopical evaluations—cell-treated scaffolds revealed greater staining with safranin, although an empty defect also showed better results than non-cell-treated scaffolds. The scaffold with chondrocytes represented the best score when the scaffolds were evaluated with the Mankin histological grading scale. However, as in previous *in vivo* evaluations, cell-treated scaffolds showed better results than non-cell-treated scaffolds. In conclusion, we have investigated that an ozone-treated scaffold containing TGFβ3 with rMDSC is a proper combination and could be a promising scaffold for cartilage regeneration.

## 1 Introduction

The subsequent healing of articular cartilage remains a significant clinical problem. Adult human articular cartilage is approximately 2–4 mm and serves as a cushion for joints against a physiological load (Hunziker et al., 2002). Because cartilage lacks nerves, blood vessels, and lymphatics, its ability to regenerate itself is restricted (Li et al., 2022). Thus, cartilage degradation can quickly lead to gradual tissue deterioration, persistent joint pain, dysfunction, and finally to the degenerative disease, osteoarthritis (OA) (Borrelli et al., 2019). To protect the cartilage from further degradation, it is necessary to apply the appropriate treatment. For many years, orthopedic surgeons treating articular cartilage injuries sought to achieve stable fixation of the articular cartilage surface restoration of limb alignment and joint stability (Borrelli et al., 2019). However, such treatment is not fully effective in cartilage preservation as it is also important to emphasize that there is an ongoing cellular response that needs to be controlled (Li et al., 2022). Following initial cartilage damage, local cells release inflammatory factors into the synovial fluid, which surrounds the cartilage (Sokolove and Lepus, 2013). These inflammatory factors, such as interleukin-1 or/and 6 (IL-1, IL-6), and tumor necrosis factor-alpha (TNF-α) inhibit chondrogenesis (Choukair et al., 2014). To improve neo-tissue formation, the impact of inflammation on cartilage tissue should be considered. A variety of techniques exist in articular cartilage repair and regeneration, each with its own advantages and drawbacks (Makris et al., 2015). The microfracture technique has limitations in chondrogenesis as this technique results in fibrocartilage formation, that is, biochemically and biomechanically inadequate for hyaline articular cartilage (Bae et al., 2006). Tissue engineering approaches using a variety of cell sources including autologous, allogenic, and xenogeneic stem cells have resulted in the repair of tissues with hyaline-like properties (Jelodari et al., 2022). Cartilage engineering by embedding relevant cells like articular chondrocytes or mesenchymal stem cells (MSCs) and growth factors, particularly transforming growth factor beta (TGFβ) (Wang et al., 2014), into scaffolds support chondrocyte growth and proliferation (Francis et al., 2018). In addition, MSCs scaffolding together with loaded differentiation factors, TGFβ1 or TGFβ3, may enhance articular cartilage formation because of their anti-inflammatory potential (Yoshimura et al., 2010; Stewart et al., 2018). MSCs are multipotent progenitor cells that can self-renew and differentiate into cartilage (Gomez-Salazar et al., 2020), while the most commonly used adult source tissue for human MSCs are bone marrow, muscle, and adipose tissue (Pittenger et al., 2019). Muscle-derived stem cells (MDSCs) have clonogenicity and growth kinetics superior to bone-derived stem cells (Čamernik et al., 2019). The necessity for a biomaterial scaffold to stimulate cell attachment, spreading, migration, proliferation, and differentiation for successful tissue regeneration is a fundamental challenge in tissue engineering in 3D microenvironments (Mohammadinejad et al., 2020). Biomaterials should be biodegradable and should have the same mechanical properties as native cartilage. However, no single best material is available that would be the gold standard for tissue engineering. Polycaprolactone (PCL) has recently received significant attention due to its mechanical strength, low immunogenicity, elasticity, biodegradability, and biocompatibility (Theodoridis et al., 2019). PCL scaffolds can promote stem cell differentiation and proliferation, while their hydrophobic profile inhibits cellular attachment, limiting their suitability in tissue engineering (Sousa et al., 2014). However, the surface of porous 3D PCL scaffold modification improves the hydrophilic properties and growth factor release (Qin et al., 2022). Treatment with ultraviolet irradiation and ozone (O_3_) increases the surface hydrophilicity of PCL scaffolds for effective cell attachment and proliferation (Samsudin et al., 2017).

In this study, we aimed to evaluate the formation of cartilage tissue using rabbit muscle-derived stem cells (rMDSCs) on electrospun bilayer PCL ozone-treated scaffolds with loaded TGFβ3. The latter has been developed and validated *in vitro* during our earlier investigations (Dabasinskaite et al., 2022; Jankauskaite et al., 2022). First, we investigated the scaffold’s potential *in vitro* to provide and maintain a microenvironment for rMDSC proliferation and differentiation into chondrocytes. In addition, to elucidate the advantages of ozone-treated (O) over non-ozonated (NO) scaffolds in order to select a more suitable scaffold variant for *in vivo* studies, we hypothesized that PCL ozone-treated scaffolds with loaded TGFβ3 and rMDSCs would outperform other groups and will show similar results to the control group—scaffolds with chondrocytes in the *in vivo* rabbit model for supporting neocartilage formation. Such demonstration of this type of scaffold has not been described earlier.

## 2 Materials and methods

### 2.1 Polycaprolactone scaffold characteristics

The scaffold was formed from a two-layer composite of fibrous matrixes, each having variations in composition and processing. The chondral layer polymer solution was composed of poly(e)caprolactone (PCL, IUPAC name: (Hunziker et al., 2002; Bae et al., 2006):-polyoxepan-2-one, CAS: 24980–41–4, Mn—80 kDa, Cat. No: 440744) dissolved with cellulose and cellulose acetate (CA, 39.7% acetyl content, Mn–50 kDa, CAS: 9004-35-7). The subchondral layer polymer solution was prepared similarly to the chondral layer, except that hydroxyapatite (HA, <15 µm particle size, CAS: 1306-06-5) powder was added. The fabrication procedure of both layers and electrospinning and ozonation techniques (treatment with O_3_) were described previously (Dabasinskaite et al., 2021; Dabasinskaite et al., 2022). PCL pellets and CA powder (2:1, w/w) were dissolved in acetone and N.N-dimethylformamide mixture at 2:3 (v/v) to obtain 30% (w/v) polymer solution. The mixing process was carried out at 40°C on the magnetic stirrer. The scaffolds were fabricated by using a cryo-electrospinning setup (voltage 26–28 kV, temperature 35°C, and RH 30%). The scaffolds were post-treated to convert cellulose acetate to cellulose and to introduce functional groups for the binding of the growth factor. The fabricated fibrous mats were cut into scaffold specimens (6 mm in diameter and a weight of 0.50 g). The scaffolds were placed in a glass reactor containing water (20°C) and treated by bubbling O_3_ from an *ex situ* generator into the reactor at a mass flow of 400 mg/h. Subsequently, the samples were stored in a vacuum dryer at 21°C for 12 h. Before *in vitro* and *in vivo* experiments, scaffolds were sterilized with ethylene oxide. The scaffold was cut into 6 mm diameter and 1 mm height sample discs for *in vitro* and *in vivo* studies.

### 2.2 Transforming growth factor-beta 3 loading on bilayer polycaprolactone scaffolds

Dosage and timing of TGFβ3 (Thermo Fisher Scientific, United States) loading on PCL were selected, as previously described (Jankauskaite et al., 2022). In brief, scaffolds were incubated with 10 ng/ml TGFβ3. After the most efficient binding duration was clarified, samples for *in vitro* and *in vivo* experiments were covered with 100 μl of 10 ng/ml TGFβ3 for 24 h, providing sufficient time for protein binding to unmodified and modified scaffolds (Dabasinskaite et al., 2022). Subsequently, the unbound protein was washed with PBS, and scaffolds were submitted for further experiments.

### 2.3 Ethics in animal experimentation

Tissue collection for rMDSC and rabbit chondrocyte (rCh) isolation and further *in vivo* studies in rabbits were approved by the Ethics Committee of the State Food and Veterinary Service (No G2-133).

### 2.4 Isolation, differentiation, and characterization of rabbit muscle-derived stem cells

Biopsies of skeletal muscles of New Zealand rabbits (*n* = 4) were collected, and rMDSCs were isolated using a pre-plating technique with some modifications (Lavasani et al., 2013). After washing, dissecting from residual tendon, fat, and connective tissue, and mechanical digestion, minced muscle tissue was submitted for enzymatic digestion with 0.2% of collagenase type XI and kept with gentle continuous rocking at 37°C for 1.5 h. Furthermore, the previously described protocol was followed with an adjusted centrifugation speed of 1,400 rpm for 5 min (Lavasani et al., 2013). Obtained cells were cultivated in a monolayer on the collagen-coated surface using Dulbecco’s modified Eagle’s medium (DMEM) with 4.5 g glucose/L (Gibco, United Kingdom), supplemented with 10% fetal bovine serum (FBS) (Gibco, United Kingdom), 10% horse serum (HS) (Gibco, United Kingdom), 0.5% chicken embryo extract (LSP, United Kingdom), and 1% penicillin/streptomycin [Penicillin/Streptomycin (10000 U/l) (P/S), Gibco, United Kingdom] at 37°C in a 5% CO_2_ humidified incubator. The medium for rMDSCs was changed every 3 days. Passages 5 up to 10 were used for further experiments.

First, the morphology of cells was evaluated *via* light microscopy. The lineage of isolated rMDSCs was identified by flow cytometry for strain biomarkers and by differentiation ability. Also, the sixth passage of rMDSCs was tested for CD45, CD44, and CD105 (Invitrogen, United States) by flow cytometry [FACSMelody (BD)]. Multipotent differentiation capacity was proven by induced, adipogenic, osteogenic, and myogenic differentiation in a monolayer and chondrogenic differentiation in pellet culture. Adipogenesis was induced with an adipogenesis differentiation medium (Gibco, United Kingdom). After 14 days of cultivation, adipogenic cultures were stained with Oil Red O (Sigma-Aldrich, United States) for microscopic visualization of lipid droplets. Osteogenesis was evaluated *via* microscopy when cultures were stained with alizarin red (Sigma-Aldrich, United States) and after 14 days of incubation with an osteogenesis differentiation medium (Gibco, United Kingdom). The chondrogenic capacity was proved by successful chondrogenic pellet formation after 21 days of incubation with chondrogenesis differentiation medium (Gibco, United Kingdom) and stained with safranin O (Sigma-Aldrich, United States) and toluidine blue (Sigma-Aldrich, United States). Myogenesis was analyzed by muscle-specific desmin protein detection with immunohistochemistry using rabbit anti-desmin antibodies (Sigma-Aldrich, United States), and cell nuclei were highlighted by DAPI fluoroshield (Sigma-Aldrich, United States).

After differentiation, rMDSCs were fixed with 1% PFA and stained, as previously described. Fixed differentiated rMDSCs were evaluated *via* microscopy (Olympus BX63, Japan).

### 2.5 Isolation and monoculture of rabbit chondrocytes

Each biopsy sample of rabbit articular cartilage was taken from the intercondylar notch and immersed in a 50-ml conical sterile polypropylene centrifuge tube (TPP, Switzerland) containing a total of 15 ml transport medium with 12 ml of DMEM, 3 ml of fetal bovine serum (FBS) (Gibco, United Kingdom), and 15 µl of 0.1% gentamycin (Gibco, United Kingdom). The biopsy was transported immediately to the laboratory for further chondrocyte isolation procedure. The sample of articular cartilage was three times washed with Hams/F12 (Gibco, United Kingdom) containing 1% penicillin/streptomycin [penicillin/streptomycin (10000 U/l), Gibco, United Kingdom]. The cartilage was then minced with a sterile scalpel blade into small pieces in a petri dish containing 2 ml of protease (type XIV, >3.5 U/mg, Sigma-Aldrich, United States) solution. The minced cartilage was transferred into the tube with 8 ml of protease solution and left for digestion for 60 min at 37°C, with 5% CO2. After 60 min, the protease solution was changed to 10 ml of collagenase (type A > 150 U/mg, Worthington Biochemical, United States) solution, and cartilage pieces were digested for 16 h at 37°C and 5% CO_2_. The enzymatic reaction was neutralized with 10 ml of DMEM supplemented with 10% FBS and 0.1% gentamycin (medium) and then filtered through a 70-µm cell strainer (Falcon, United Kingdom) into a 50-ml tube and centrifuged at x 300 g (4°C) for 10 min. The supernatant was carefully discarded, and the cell pellet was filled with a fresh 1 ml of medium. The mixture was resuspended, and the cell count and viability were determined by the trypan blue dye exclusion test. The cells were plated in tissue culture flasks at a density of 2 × 10^3^ cell/cm^2^. The morphology of the cells was examined regularly, and the image was taken with a microscope. When the cells reached 80%–90% confluence, they were trypsinized by TrypLE-Express enzyme (x1) and phenol red (Gibco, United Kingdom), and chondrocytes [passage 0 (P0)] were harvested, centrifuged, and resuspended in a culture medium. The culture medium was changed every 3 days. Cells were replated at a density of 2−6 × 10^3^ cm^2^.

### 2.6 Pellet culture

rMDSC and rCh pellets were established in microcentrifuge tubes by suspension of 2 × 10^5^ cells in 400 µl of the medium. The tubes were centrifuged at x 300 g (4°C) for 6 min. The tops of the tubes were perforated with an 18-gauge needle after centrifugation to permit gaseous exchange. After 72 h, corresponding to the first time, the medium was changed, and the pellets were gently detached from the bottom. This procedure was repeated every 3 days until the 21-day pellet-culturing time-point was reached. The rMDSC pellet was cultured in a chondrogenesis differentiation medium (Gibco, United Kingdom). Control pellets containing rMDSC cells were cultured using an identical cell culture medium described in Section 2.5. The triplets of both pellets including rMDSC and control were formed.

### 2.7 Cell proliferation assay

Unstained cell-scaffold complexes were cultivated in 96-well plates. Briefly, cells were seeded on scaffolds at a density of 50,000 cells per scaffold. Three replicates were set up for each group. Cell proliferation was examined at the following time-points: days 1, 3, 7, 14, and 21. On each day, 10 μl of the CCK-8 (Abcam, United States) solution was added to each well with the cell-scaffold complex. After 3–4 h incubation, the medium was collected into separate 96-well plates, and the absorbance of each well was measured using a microplate reader (Thermo Fisher Scientific, Finland).

### 2.8 Enzyme-linked immunosorbent assay

TGFβ3 and collagen 2 (Coll2) concentrations in the supernatants during culturing were quantified using ELISA kits. The cell culture medium from TGFβ3-loaded NO or O scaffolds and non-TGFβ3-loaded NO or O scaffolds, both rMDSC and hCh were aspirated on days 1, 3, 7, 14, 21, and 28, frozen at −80°C, and kept until needed (less than one month). TGFβ3 and Coll2 were measured according to the protocols of commercially available ELISA kits: TGFβ3 (Assay Biotech, United States) and Coll2 (Cloud-Clone Corp., United States). Absorption at 450 nm was measured using a microplate reader (Multiskan GO 1.00.40 (Thermo Fisher Scientific)).

### 2.9 Animal surgery and scaffold preparation

**FIGURE 1:**
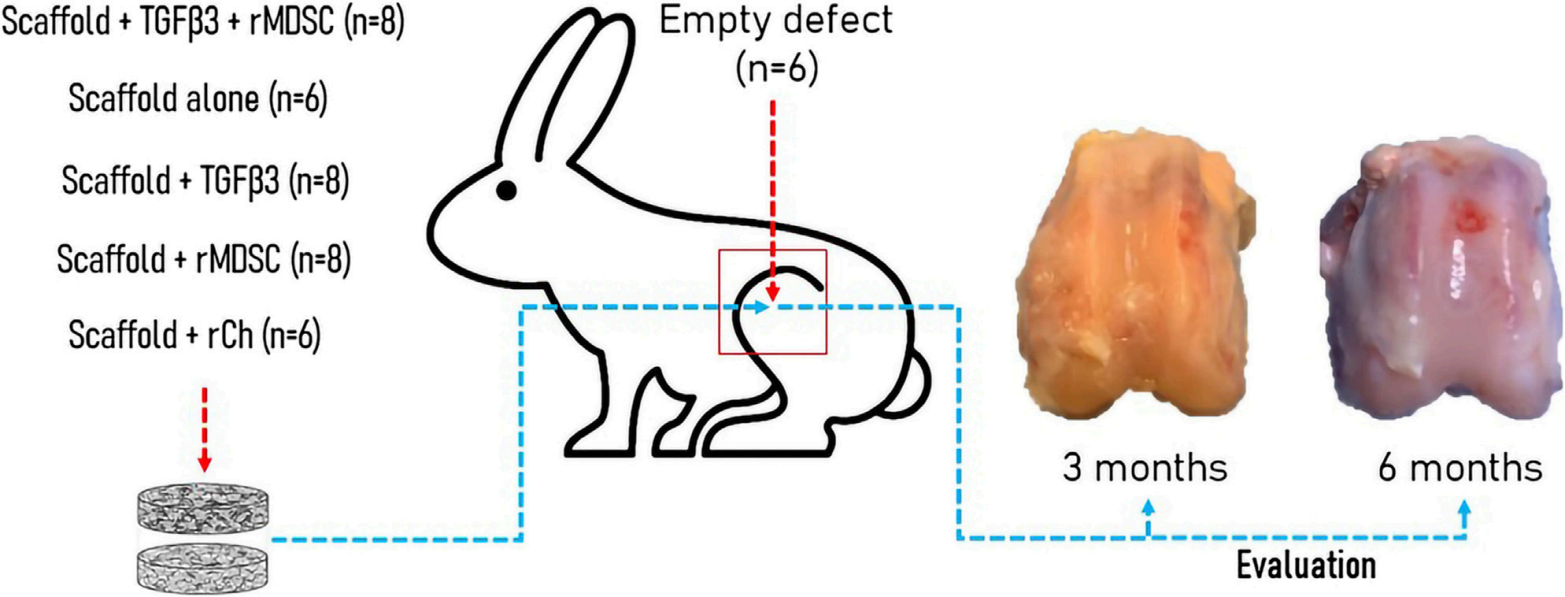
Experimental *in vivo* rabbit model. Different groups were formed according to the type of implant into the cartilage defect. Evaluation of cartilage repair including macroscopic, electromechanical, and histological analyses was performed after 3 and 6 months. TGFβ3, transforming growth factor-beta 3; rMDSCs, rabbit muscle-derived stem cells; rCh, rabbit chondrocyte.

Twenty-three male 3–4-month-old New-Zeeland White rabbits were used for the study. Each knee was randomly assigned to one of the following treatment groups. There were six experimental groups according to the treatment and bilayer scaffold preparation for implantation into cartilage defect (Figure 1). There were the following groups: empty defect (E, *n* = 6), scaffold alone (cell-free and growth factor free) (S, *n* = 6), TGFβ3-loaded scaffold (St, *n* = 8), TGFβ3-loaded scaffold + rMDSC (Stm, *n* = 8), scaffold with rMDSC (Sm, *n* = 8), and scaffold with rabbit chondrocyte (rCh) (Sc, *n* = 6) groups. Ozonated scaffolds were used in all groups. Rabbits were euthanized 3 and 6 months after surgery. Two animals did not survive until the end of the designated time-point and were excluded from the study.

### 2.10 Surgical procedure

Under general isoflurane (Vetpharma Animal Health, Barcelona, Spain) anesthesia, following shaving and sterile preparation of both legs, a 3-cm medial parapatellar incision was made in each knee, exposing the patellofemoral groove. Using slow-speed dental trephine under constant irrigation with saline, an osteochondral defect, measuring 4.5 mm in diameter and 4–5 mm deep, was created in a trochlear groove in both knees. Defects were filled with a two-layer scaffold that consisted of the chondral layer made of poly(*e*)caprolactone and cellulose (PCL-CEL) at the top prepared in different ways and the subchondral layer made of PCL-CEL-HA at the bottom. Scaffolds were press-fitted into the cartilage defect and sealed with fibrin glue (Tisseel Lyo, Baxter, Switzerland). Following wound closure, the knee was moved through a full range of motion to ensure normal patellar tracking. After surgery, all animals were allowed to move freely in the cages and provided with food and water *ad libitum*.

### 2.11 Macroscopic evaluation and grading of the cartilage repair site

Thereafter, 3 and 6 months after implantation, animals were euthanized, and the articular cartilage defect repair site was evaluated macroscopically and assessed using a modified Oswestry Arthroscopy Score (OAS) by two independent researchers. According to this scoring system, the score was based on a point system with a total of 8 points, representing healthy cartilage. Repair tissue surface level, integration with surrounding cartilage, appearance, and color of the repair tissue surface were assessed. Stiffness on probing was excluded in our study because repaired cartilage properties were evaluated electromechanically by using an Arthro-BST device (Biomomentum Inc., Laval, Quebec, Canada).

### 2.12 Electromechanical evaluation

Electromechanical properties of cartilage were evaluated using an Arthro-BST device 3 and 6 months after transplantation, as described elsewhere (Mickevicius et al., 2015). Briefly, positively charged mobile ions in the cartilage stroma are displaced, with respect to the fixed and negatively charged proteoglycan molecules during cartilage compression. The probe of the device registers streaming potentials after compression, and with the assistance of software, it generates quantitative parameters (QPs) in numeric values from 0 to 36. The high QP parameter is a digital reflection of extracellular matrix disintegration, weak electromechanical properties, and inferior load-bearing capacity of the cartilage, while low QP indicates strong electromechanical properties and superior load-bearing capacity. QP measurements in each repair site were recorded four to five times to obtain median values.

### 2.13 Histological evaluation and grading

After macroscopic and QP examination, distal femurs of the rabbits were dissected, fixed in 10% neutral buffered formalin, decalcified, and embedded in paraffin. Then, 5-F06DM thick sagittal sections were stained with safranin O/fast green, as previously described (Mickevicius et al., 2015). Light microscopy images of the repair site were taken at x 40 magnification with an Olympus BX61 microscope equipped with an Olympus DP72 CCD camera by cellSens Dimension imaging software (Olympus, Japan). A total of 30 snap pictures of the entire implantation site were combined into one final picture using the manual stitched image acquisition function of the software. The quality of the repaired cartilage was blindly evaluated by two investigators using the Mankin histological grading score, as described in Pearson et al. (2011). According to the Mankin grading system, healthy cartilage is rated with 0 points. The articular surface is rated 0–4 from smooth to cracked or completely disorganized, respectively. Chondrocyte morphology and proliferation are assessed from 0 (normal cell distribution) to 3 points (zones without cells). The extent of safranin-O staining is assessed from 0 to 4 points (not stained). With a maximum of 11 points, the cartilage is estimated as fully damaged.

### 2.14 Statistical analysis

Statistical analysis was accomplished by IBM SPSS 28.0 software (SPSS Inc., Chicago, IL, United States) for Windows. All the quantitative data were expressed as mean ± standard deviation (SD). Kruskal–Wallis and Mann–Whitney U tests were used to analyze the differences. A *p*-value of <0.05 was considered significant.

## 3 Results

### 3.1 Isolation, differentiation, and characterization of rabbit muscle-derived stem cells

**FIGURE 2:**
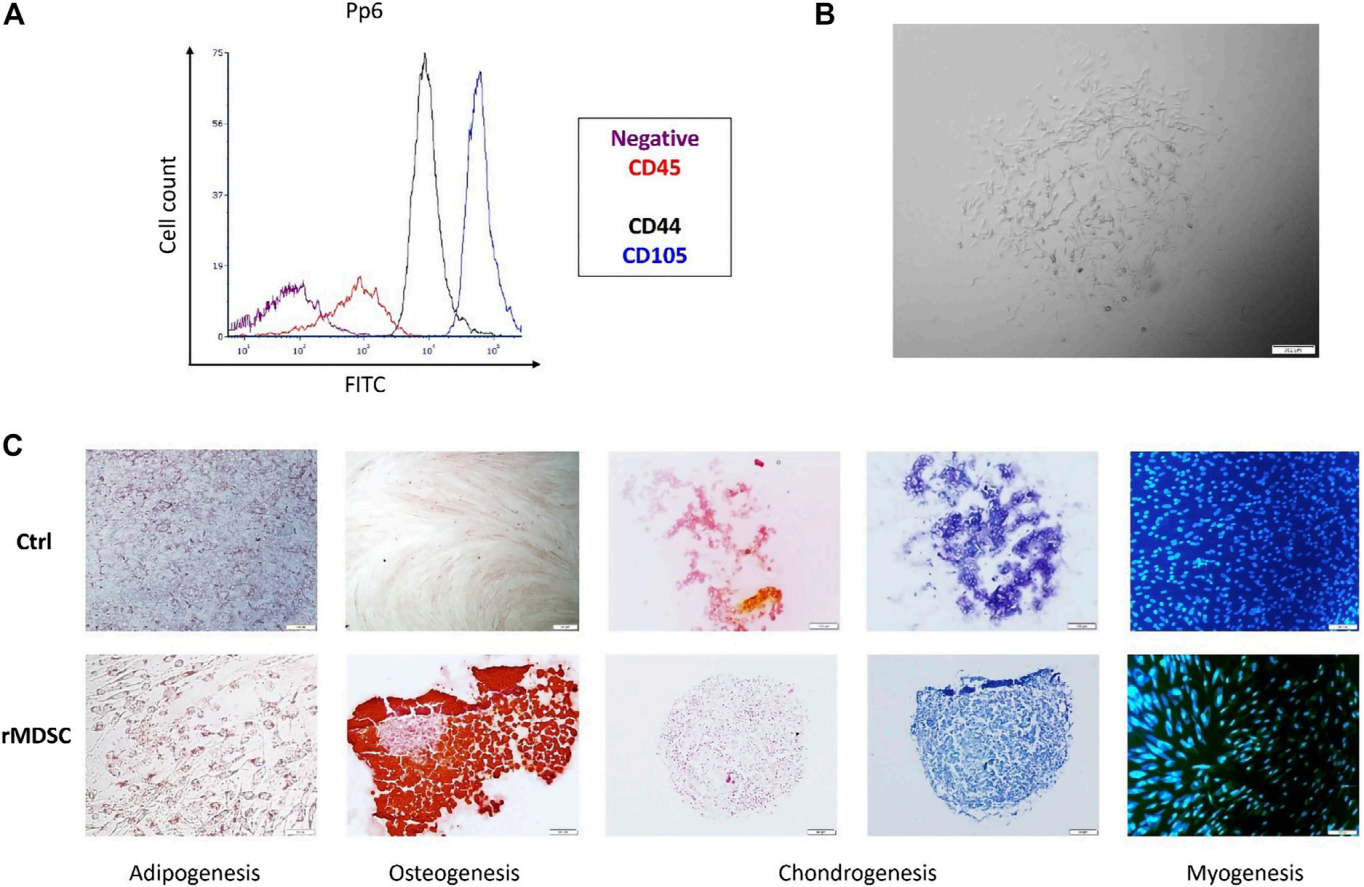
Characterization of isolated rMDSCs. The expression of specific stem-cell markers of preplate 6 (pp6) was tested *via* FACS **(A)**. Pp6 was differentiated into adipocytes, osteocytes, chondrocytes, and myocytes **(C)**. Isolated cell (preplate 6; passage 5) morphology was identified *via* microscopy **(B)**. **(A)** purple color—negative control, red—CD45, black—CD44, and blue—CD105; **(C)** adipogenesis—Oil Red O staining; osteogenesis—Alizarin red staining; chondrogenesis—safranin O (orange-red) and toluidine blue (blue) staining; myogenesis—DAPI (blue) and desmin (Fitc). CD, cluster of difference; pp6, preplate 6; rMDSCs, rabbit muscle-derived stem cells; ctrl, undifferentiated rMDSCs. Scale bar –100 μm.

Rabbit MDSCs were isolated from a few rabbits and analyzed by flow cytometry. FACS analysis showed the expressions of rabbit stem cell surface markers (CD44 and CD105) and lack of the hematopoietic marker (CD45) on pp6 (Figure 2A). Isolated cells excelled by homogeneity and rapid proliferation after pre-plate 6 cells were expanded (Figure 2B). Pp6 cells demonstrated adipogenic differentiation capacity after 14 days of cultivation in an adipogenic induction medium (Figure 2C, adipogenesis). The myogenic potential was determined by finding desmin-positive cells after 18 days of incubation with a low-serum myogenic medium (Figure 2C, myogenesis). After 14 days, rabbit MDSCs differentiated into osteocytes, and after 21 days, they formed a chondrogenic pallet which stained positive for safranin and toluidine blue, as shown in Figure 2C.

### 3.2 Rabbit muscle-derived stem cell proliferation and differentiation into chondrocytes

**FIGURE 3:**
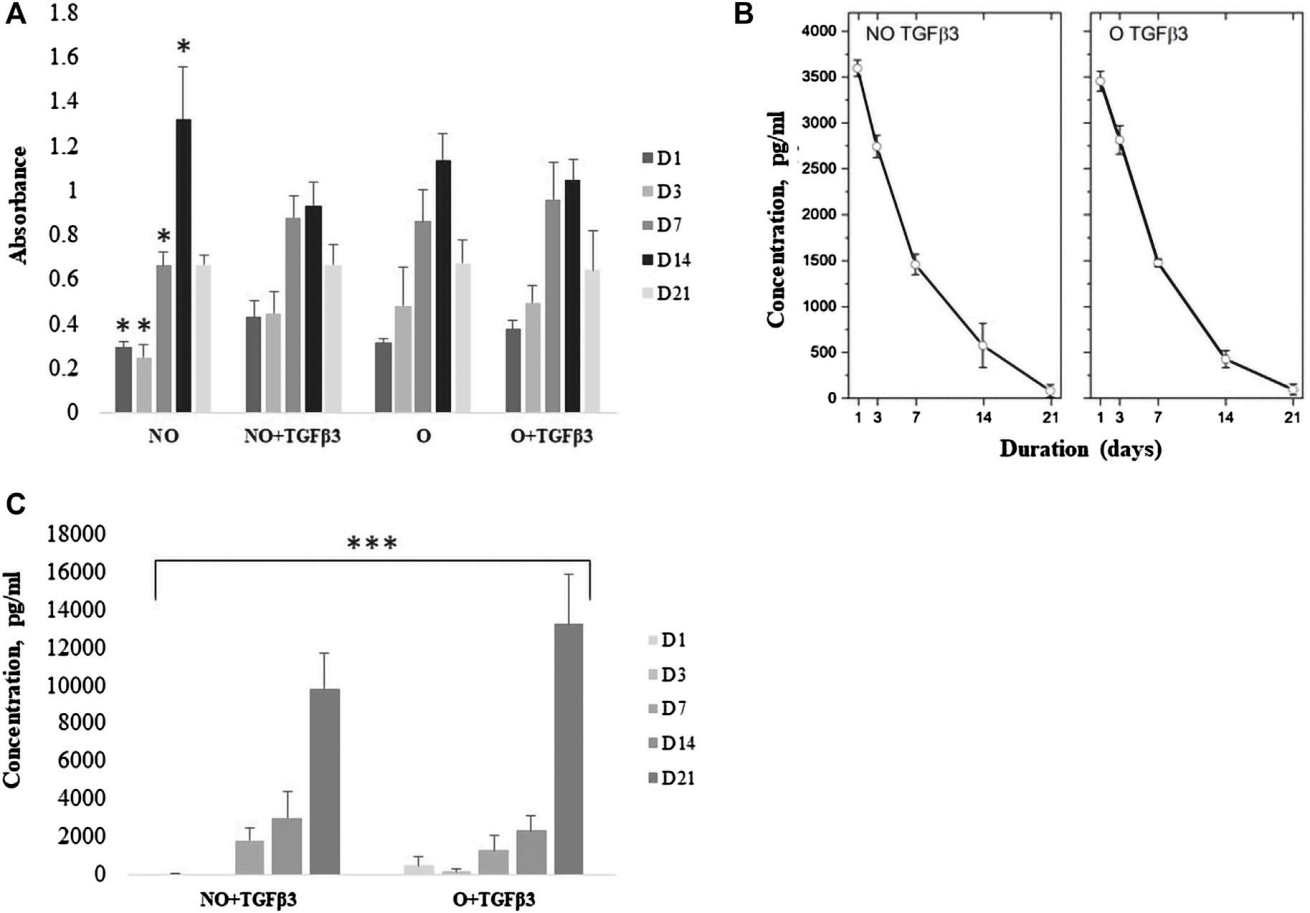
Cell proliferation within scaffolds **(A)**, TGFβ3 release from scaffolds with rMDSCs **(B)** and collagen-II (Coll2) protein production from ozone-treated and untreated scaffolds with loaded TGFβ3 **(C)**. The cell proliferation rate was tested on days **(D)** 1, 3, 7, 14, and, 21 *via* CCK-8. TGFβ3 and collagen-II protein levels were tested at the same time-points *via* ELISA. Results are presented as mean ± standard deviation. * represents *p*-value <0.01 comparing NO at D1, 3, and 7 to NO loaded with TGFβ3 and O with or without TGFβ3. *p*-value <0.01 also belongs to NO D14 compared with NO loaded with TGFβ3 **(A)**. *** represents <0.001 *p*-value; the Kruskal–Wallis test showed a significant difference between multiple time-points (D1–D21) compared to NO and O scaffolds loaded with TGFβ3 in Coll2 protein analyses **(C)**. NO-untreated; O, ozone-treated; TGFβ3, transforming growth factor-beta 3; pg/ml, picograms per milliliter.

At the initial time-points (D1–D7), there was a significantly lower proliferation rate of rMDSCs on NO scaffolds than NO loaded with TGFβ3, as well as O with or without a TGFβ3 (*p* < 0.01) (Figure 3A). On day 14, NO showed a significant increase in rMDSC growth compared to NO loaded with TGFβ3. However, there were no significant cell growth changes when compared to the other two scaffolds. We analyzed the TGFβ3 protein release but observed no difference between the NO and O scaffolds loaded with TGFβ3 (*p* > 0.05) (Figure 3B). In addition, untreated scaffolds were tested to assess if rMDSCs produced TGFβ3; however, no protein was identified (data not shown).

Nevertheless, on day 21, ozonated TGFβ3-loaded PCL scaffolds showed increased Coll2 production, suggesting rMDSC differentiation into chondrocytes (*p* < 0.001) (Figure 3C).

### 3.3 Macroscopic evaluation and grading of the cartilage repair site

Considering that ozonated scaffolds provided a better environment in our *in vitro* studies and based on our previous research (Jankauskaite et al., 2022), they were further submitted for *in vivo* experiments.

**FIGURE 4:**
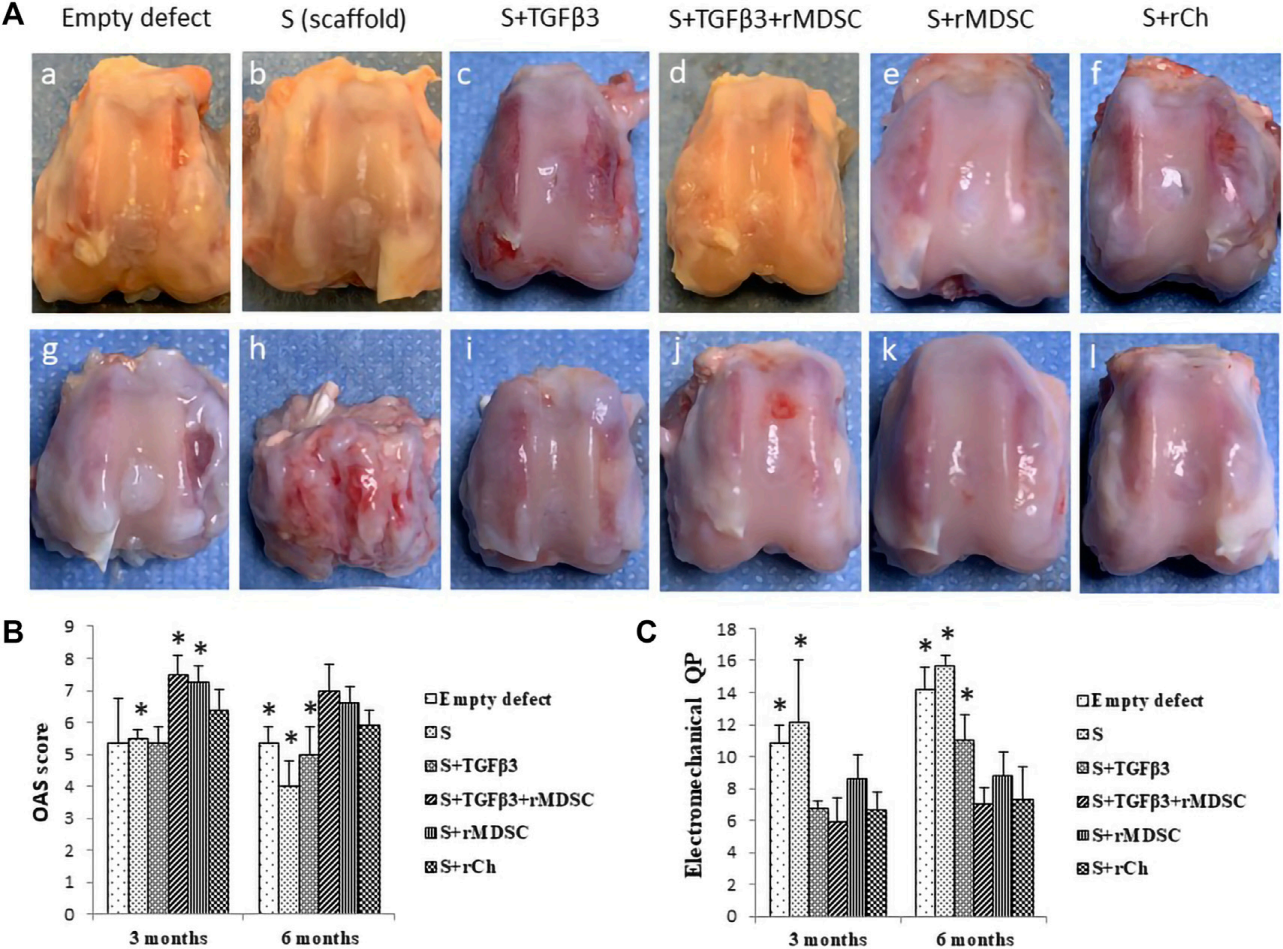
Healing of cartilage defects with different scaffolds after 3 **(A–F)** and 6 (g, h, i, j, k, and l) months **(A)**. Macroscopic evaluation of cartilage defect healing according to the Oswestry Arthroscopy Score (OAS) **(B)**. Evaluation of cartilage electromechanical quantitative parameters (QPs) by using the Arthro-BST device **(C)**. Results are presented as mean ± standard deviation. * represents *p*-value <0.05 compared to Sc with other scaffolds—S, Stm, and Sm at 3 months. In addition, the *p*-value <0.05 represents the difference compared to Sc with E, S, and St at 6 months **(B)**. *p*-value <0.05 showed as * indicates significant differences between Sc and E or S scaffolds at 3 months, also comparing Sc with E, S, and St scaffolds at 6 months **(C)**. S, scaffold; TGFβ3, transforming growth factor-beta 3; rMDSCs, rabbit muscle-derived stem cells; rCh, rabbit chondrocyte. Abbreviations of the scaffolds (E, S, St, Stm, Sm, and Sc) are described in Section 2.9.

The macroscopic images of the rabbit femoral condyles at 3 and 6 months after transplantation are depicted in Figure 4A. The mean Oswestry Arthroscopy Score (OAS) values in each experimental group are shown in Figure 4B.

Stm and Sm demonstrated better results 3 months after transplantation (mean score—7.50 ± 0.58 and 7.25 ± 0.5, respectively). Stm and Sm had significantly higher scores than E, S, St, or Sc. Sc showed significantly higher scores (mean score 6.38 ± 0.63) than S but significantly lower scores than Stm and Sm. Overall, E, S, and St had lower scores (5.38 ± 1.38, 4.88 ± 0.25, and 5.38 ± 0.48, respectively).

At 6 months post-transplantation, Stm scored a high score (7.0 ± 0.82), and Sm and Sc scores were significantly higher than E, S, and St, but no difference was found between these groups. Sc was superior to E, S, and St (*p* < 0.05) (Figure 4B).

Overall, during the period from 3 to 6 months, results tended to show slight macroscopic score deterioration. Defects filled with S and Sc had statistically lower scores at 6 months than at 3 months, while scores of E, St, Stm, and Sm were similar between the time-points.

### 3.4 Electromechanical evaluation

Greater electromechanical quantitative parameter (QP) values represent greater degenerative changes in the cartilage (Figure 4C). After 3 months post-transplantation, the highest QP values were observed in the empty defect € and defect filled with scaffold alone (S) (10.85 ± 1.1 and 12.5 ± 3.87, respectively) compared to defects filled with St, Stm, and Sc scaffolds which had significantly lower QP values *p* < 0.05. St-, Stm-, and Sm-treated groups revealed similar results to Sc. Overall, Stm demonstrated the best QP value (5.95 ± 1.42); however, it was not significant compared to St, Sm, and Sc.

We observed similar tendencies 6 months post-transplantation. Worst QP values were registered in the E and S groups (14.15 ± 1.46 and 15.69 ± 1.46, respectively). In contrast to 3 months, at 6 months post-transplantation, S and St had significantly worse results than scaffolds with cells (*p* < 0.05). The best electromechanical values were attributed to Stm- and Sc-treated groups (7.05 ± 1 and 7.30 ± 2.04, respectively).

Comparing degenerative processes between 3 and 6 months post-transplantation, scaffolds without cells demonstrated clearly worse results than cellular scaffolds. The worst electromechanical measurements were found in empty defects and S scaffolds (Figure 4C). However, no statistical cartilage deterioration was observed in all cellular scaffolds after 6 months.

### 3.5 Histological evaluation and grading

**FIGURE 5:**
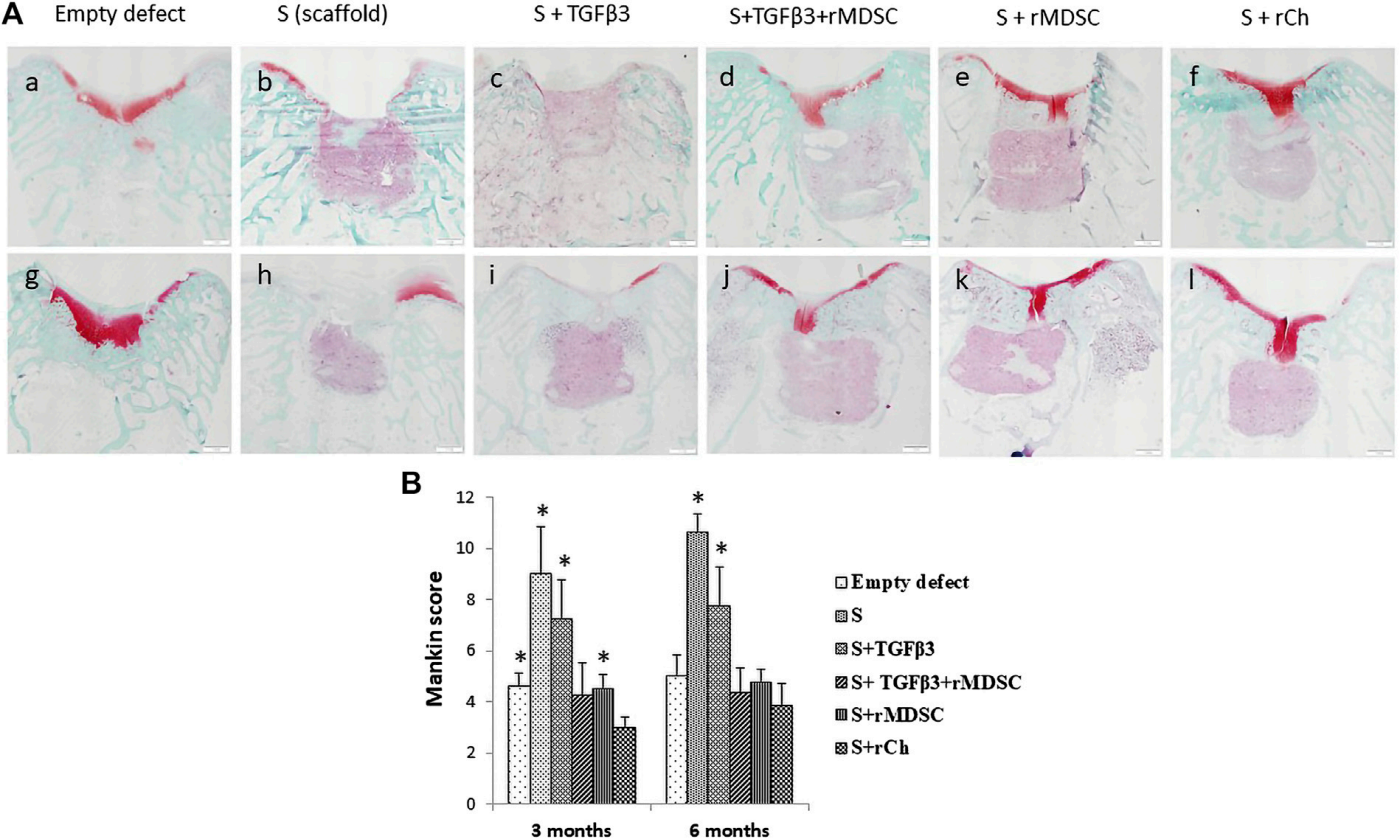
Safranin O staining of cartilage defects in different treatment groups after 3 **(A–F)** and 6 (g, h, i, j, k, and l) months **(A)**. Evaluation of cartilage healing according to the Mankin histological grading scale **(B)**. Scale bar-1 mm. Results are presented as mean ± standard deviation. * represents *p*-value <0.05 Sc with other scaffolds, E, S, St, and Sm scaffolds at 3 months. In addition, the *p*-value <0.05 represents a statistically significant difference compared to Sc with S and St scaffolds at 6 months. S, scaffold; TGFβ3, transforming growth factor-beta 3; rMDSCs, rabbit muscle-derived stem cells; rCh, rabbit chondrocyte. Abbreviations of the scaffolds (E, S, St, Stm, Sm, and Sc) are described in Section 2.9.

Images of histological sections stained with safranin-O/fast green are presented in Figure 5A. Mankin histological grading scores in each experimental group are summarized in Figure 5B. At 3 months postoperatively, the repaired tissue was consistently well organized in all groups. Membranes were partially bonded to the adjacent cartilage and the subchondral bone, and little clustering was detected at the border of native cartilage. Active integration with adjacent cartilage was less evident. All grafts exhibited hyaline cartilage morphology (scarcity of safranin-O staining). Sc scaffolds demonstrated the best results after 3 months (3.0 ± 0.41). Stm and Sm had similar scores and were significantly better than S and St scaffolds (4.25 ± 1.26 and 4.5 ± 0.58, respectively). Interestingly, inferior results were observed in S (9.0 ± 1.83) and St scaffolds (7.25 ± 1.5) compared to cellular scaffolds (*p* < 0.05). Remarkably, empty defects had significantly better scores than S and St scaffolds and significantly worse scores than Sc scaffolds (*p* < 0.05), but no difference was observed with Stm and Sm scaffolds.

We observed similar results after 6 months. E, St, Stm, and Sm scores were similar. The worst results were observed in S and St scaffold groups (10.63 ± 0.75 and 7.75 ± 1.5, respectively), and it was clearly inferior to all other treatment groups (*p* < 0.05).

## 4 Discussion

With an increased life expectancy, there is an augmentation in degenerative conditions affecting cartilage tissue; thus, articular cartilage repair *via* different regenerative techniques is highly investigated. Recently, a focus has been set on engineering a biomimetic cellular microenvironment for the regeneration of a cartilaginous tissue, which has no potential to rejuvenate by itself. In our study, we aimed to examine the properties of an electrospun PCL bilayer scaffold consisting of a chondral layer with or without TGFβ3 and seeded with rMDSC and a subchondral layer supplemented with HA. To the best of our knowledge, this is the first study demonstrating that the PCL scaffold created a proper microenvironment for rabbit MDSCs and showed highly promising results toward neocartilage formation *in vitro* and *in vivo*.

Cells and their microenvironment are highly important in the regenerative process. First, such elements as chemical properties, technical composition, and mechanics are associated with cell behavior, e.g., their migration, proliferation, or differentiation (Demoor et al., 2014; Girão et al., 2020; Abpeikar et al., 2021). For our scaffold production, we selected PCL, a highly biodegradable, elastic, and low cytotoxic material used in the electrospinning process, which provided a porous structure for our scaffolds. The porosity of the chondral layer was 90.7%; the mean pore diameter was 171 µm. For the subchondral layer, porosity was 94.4%, and the mean pore diameter was 240 µm. To improve its properties, scaffolds underwent ozone treatment and were further modified by supplementation of TGFβ3 protein. Ozone treatment has been already analyzed and shown to impact cell adhesion and proliferation (Rediguieri et al., 2017; Samsudin et al., 2018). In addition, this process can improve attachment of growth factors such as insulin growth factor-1 (Dabasinskaite et al., 2021) necessary to increase required differentiation toward chondrocytes, while the present study did not show an obvious impact on better TGFβ3 attachment than non-ozonated scaffolds. As we used rMDSCs, we selected TGFβ3 as the additional factor which is a confirmed factor in stimulating stem cells to undergo the chondrogenesis process (Grafe et al., 2018; Music et al., 2020). This material and technique already examined in different studies created a proper environment for our cells—rabbit MDSCs and chondrocytes. We did show its potential in the previous experimental study as well (Jankauskaite et al., 2022). The proliferation capacity was increased within the first 14 days. Ozonation and protein loading gave clear advancement as rMDSCs proliferated significantly more in three scaffolds (NO + TGFβ3, O, and O + TGFβ3) and provided a superior environment for the cells. Even though we could not detect differences in TGFβ3 amounts between NO and O loaded with protein, ozone-treated protein-loaded PCL scaffolds demonstrated to provide a superior environment regarding neocartilage formation *in vitro*. The environment influencing and inducing proliferation is important, but differentiation toward chondrocytes is obligatory. Our scaffolds met those two requirements; moreover, cells were viable for 21 days, and differentiation into chondrocytes was observed starting on day 7. Interestingly, ozonated scaffolds loaded with TGFβ3 stimulated chondrogenesis earlier—starting on day 1 (after seeding).

At 3 months, the histological defect filled with the scaffold loaded with TGFβ3 and seeded with rMDSC had statistically comparable regeneration to the control group—the scaffold with chondrocytes. When scaffold-containing rMDSC was compared to a scaffold loaded with TGFβ3, the previous scaffold showed better regeneration. It suggests that rMDSCs, in the presence of TGFβ3, provide superior conditions for cartilage regeneration than using scaffolds only with cells. The positive effect of TGFβ3 in promoting chondrogenesis has been shown in the literature with other stem cells (Sun et al., 2018; Li et al., 2020). Moreover, we observed better macroscopic healing results after 3 months post-transplantation in animals treated with scaffolds containing rMDSC and TGFβ3 compared to scaffolds with chondrocytes alone. After 6 months, scaffolds with rMDSC and scaffolds containing TGFβ3 had comparable histological and macroscopic results. The findings indicate the TGFβ3 effect on the rMDSC ability to regenerate cartilage tissue for up to 3 months. On the contrary, after 6 months, scaffolds with TGFβ3 had significantly better outcomes than scaffolds alone. The findings suggest that scaffolds with the addition of TGFβ3 may stimulate surrounding host cells for up to 6 months. Various studies proved the beneficial effect of TGFβ3 on cartilage regeneration using different cell types and constructs (Sun et al., 2018; Li et al., 2020; Barati et al., 2020). As our scaffold is well biodegradable, the supplementation of TGFβ3 in the scaffold can promote differentiation and proliferation of remaining tissue chondrocytes in the scenario of cartilage injury (Li et al., 2022). In addition, it is possible that TGFβ3 can engage other types of cells circulating in the synovial fluid including fibroblasts (Denu et al., 2016), MSCs (Barati et al., 2020), or even fat cells (Li et al., 2020) to form new cartilage. At 6 months, a scaffold with TGFβ3 and rMDSCs showed comparable electromechanical, macroscopic, and histological outcomes to the golden standard scaffold in our study called “control scaffold”—the scaffold embedded with chondrocytes. Chondrocyte-scaffold constructs are already widely applied in cartilage regeneration (Nam et al., 2009; Kon et al., 2011; Anders et al., 2012), having in mind the side effects of stem cells (overgrowth, tumor formation, need for specific conditions for specific tissue formation, etc.). However, the use of chondrocytes has its drawbacks because it can lead to the dedifferentiation of these cells (Mayne et al., 1976; Harrison et al., 2000; Stokes et al., 2001). This can result in further loss of tissue, increase inflammation, and cause progressing loss of the function of the joint. Therefore, another promising technique may be the use of MSCs and growth factors with optimally selected cell count and concentration of growth factor, as well as the selection of biomaterial and its appropriate fabrication to ensure the proper environment for seeded cells. In our study, over the 6-month period, the worsening results of the overall non-cellular groups were determined to show non-endurant properties of the regenerated tissue. Most of the cellular scaffolds demonstrated similar results between the same time-points. Sm and Stm scaffolds had comparable results between 3 and 6 months macroscopically, histologically, and electromechanically, indicating regenerated cartilage tissue-endurant properties over a 3-month period.

In summary, the present study showed the potential of the bilayer PCL scaffold, which was ozonated and loaded with TGFβ3 to stimulate rMDSC differentiation into cartilage tissue *in vitro* and *in vivo* in the rabbit model. This is the first study showing promising results of the combination of different factors (use of two differently prepared layers of the scaffold, ozone treatment, and embedding of stem cells) on articular cartilage regeneration. However, in order to translate the use of our developed scaffold into clinical studies, additional *in vitro* studies should be completed to eliminate the limitations of this scaffold. In addition, more *in vitro* studies should be performed to analyze the selection of the most appropriate concentration of TGFβ3 and the duration when scaffolds are incubated with the growth factor. Repeated *in vivo* studies are required if the results of *in vitro* studies will be significantly inferior/superior to those presented in our experiments. Adding, a longer *in vivo* study with regard to the articular joint function should be accomplished.

## Data Availability

The original contributions presented in the study are included in the article/Supplementary Material; further inquiries can be directed to the corresponding author.
